# Social learning through prediction error in the brain

**DOI:** 10.1038/s41539-017-0009-2

**Published:** 2017-06-16

**Authors:** Jessica Joiner, Matthew Piva, Courtney Turrin, Steve W. C. Chang

**Affiliations:** 1 Department of Psychology, Yale University, New Haven, CT 06511 USA; 2 Department of Neuroscience, Yale School of Medicine, New Haven, CT 06520 USA; 3 Interdepartmental Neuroscience Program, Yale University School of Medicine, New Haven, CT 06510 USA

## Abstract

Learning about the world is critical to survival and success. In social animals, learning about others is a necessary component of navigating the social world, ultimately contributing to increasing evolutionary fitness. How humans and nonhuman animals represent the internal states and experiences of others has long been a subject of intense interest in the developmental psychology tradition, and, more recently, in studies of learning and decision making involving self and other. In this review, we explore how psychology conceptualizes the process of representing others, and how neuroscience has uncovered correlates of reinforcement learning signals to explore the neural mechanisms underlying social learning from the perspective of representing reward-related information about self and other. In particular, we discuss self-referenced and other-referenced types of reward prediction errors across multiple brain structures that effectively allow reinforcement learning algorithms to mediate social learning. Prediction-based computational principles in the brain may be strikingly conserved between self-referenced and other-referenced information.

## Historical perspectives on representing other

Learning about the world and making adaptive decisions is a critical feature of cognition. This important link allows human and nonhuman animals to manipulate their environment and survive. Decision-making takes on more complex dynamics when an animal is not solitary, but lives in a community with other members of its own species. We know much about how human and nonhuman animals learn from their own actions and outcomes, and where such self-referenced information is represented in the brain. However, much less is known about the computations underlying how we learn about others. In this review, we examine the presence of other-referenced prediction errors in the brain that represent other’s actions and reward outcomes.

One of the first academic disciplines to attempt to understand how we develop a concept of others is developmental psychology, in which researchers often explore how babies come to understand the world.^1, 2^ One viewpoint, that of theory–theory, is that, like small scientists testing causal relations,^3^ children are constantly gathering data from the world and testing the predictions they make using the collected data. Other people could be regarded as stimuli to be learned about based on observation and direct and vicarious experience.

In contrast, simulation theory assumes that we develop our understanding of others through self-referencing, in which we use the machinery that we use for our own mental processes, and project that knowledge onto other’s behaviors.^4^ Later, this notion of simulating others became associated with the “mirroring” neuronal activity observed in individual cortical motor neurons when macaque monkeys observe an action and perform the same action.^5^

Notably, these theories have different predictions about how other-referenced information is represented in the brain.^6^ In a simulationist account, the notion of “other” is derived from one’s sense of the self, that is, egocentrically. Ideas about others would originate from and rely on the self-referenced, egocentric mechanisms. However, in theory–theory, information about others would be processed and evaluated like any other information from the environment, perhaps engaging allocentric systems. Historically, these two ideas capture a central question about how others are represented in the brain.

## Observational and social learning

Both human and nonhuman animals rely on observation to navigate the world. Rats,^7^ birds,^8^ and chimpanzees^9^ observe others to learn about their behaviors in a given environmental or social context. One of the earliest forms of observational learning occurs in imitation. Imitative learning typically involves a young organism copying the motor behaviors of a social exemplar. Infant humans^10^ and infant monkeys^11^ imitate gross facial expressions made by a caregiver early on in development, sticking out their tongue reflexively when an adult human demonstrates, likely an example of a simple motor response prepared by the brain for social development. Imitation in human children is most famously exemplified in observational learning or, more broadly, social learning studies.^12^

Social learning occurs when the learner watches another agent act. Notably, without any practice or direct primary reinforcement, the learner can perform the previously observed behavior. This suggests that the learner is able to acquire new knowledge or skills from watching other’s experienced outcomes, possibly through vicarious reinforcement.^13^ The efficacy of social learning depends on several social variables. For example, similarity between the observer and the observed increases the efficacy of learning.^14^ Furthermore, there is a close link between empathy and social learning. Empathy is sensitive to learned information about the traits of the other person, such as how fair they are^15^ or whether or not the other person is considered in-group or out-group relative to the observer.^16^ In addition, social status directly affects learning based on others in primates, in which high-status individuals are more likely to be imitated.^17^

In humans, observational learning may be at the core of establishing social and cultural norms.^12^ In Bandura’s classic study^13^ on behavioral modeling, children that saw an adult model aggressive behavior toward a large doll later performed the same aggressive behaviors when given the opportunity to interact with the same doll. Observational learning has an important role in development as well as in later social interactions and social cognition. A critical question in social learning is how self and other are represented across the brain structures involved in learning and whether the learning-related signals referenced to self and other are engaging similar or distinct neural computations.

Social learning as we define it in this review focuses on this observation-based learning, in which a subject learns about another through observing their actions as well as their reward outcomes. However, the aspects of social learning are as multitudinous as the facets of a social interaction itself. One could learn about different aspects of the other, such as their personality or their mental state. Social learning could also reflect learning from others about one’s own reward outcomes (e.g., a teacher providing feedback on a student’s essay and grade).

## Higher-level social cognition

Learning about others allows us to model the internal states of other entities. The ability to model another’s beliefs is called Theory of Mind (ToM). ToM is arguably the most complex form of understanding other individuals, heavily engaging other-referenced processing. The fact that human babies can model the beliefs of others speaks to how complex and rich their representation of the world is from the beginning. Not surprisingly, understanding the mechanisms behind ToM has long been of great interest, with competing ideas about whether or not ToM represents a separate social process or the convergence of many generalized processes.^18^ ToM is often measured using a false belief task,^19^ which tests if a participant can understand whether a social model has an incorrect belief about an object’s location. Notably, very young children, even as young as 11-month-old infants, are capable of modeling the internal beliefs of others and “pass” a false belief test,^20^ indicating that other-referenced processing in the brain emerges very early in the human ontogeny.

Studying ToM in nonhuman animals, however, has led to more mixed results. For example, monkeys fail the same false belief tasks infants can pass.^21^ Nevertheless, nonhuman primates have been shown to engage in other forms of understanding or at least representing others. Monkeys display joint attention via gaze following.^22–24^ Monkeys will typically follow the gaze of another entity toward an object or direction, indicating either that they can understand something about the perspective of the other entity or that other’s gaze is reflexively allocating one’s attention through hard-wired neural mechanisms evolved to deal with the association between other’s gaze angle and something of interest and value. Similarly, monkeys^25^ and chimpanzees^26^ have been shown to comprehend what visual information is available to a separate agent—for example, when given an opportunity to steal food, they prefer to do so from someone who does not have visual access to them at the moment of theft. This indicates that primates understand other entities have a different perspective, even if they do not necessarily model the beliefs of the other entity.

Taken together, both humans and nonhuman animals are capable of complex social cognition, but the level of sophistication is what might differentiate them in the evolutionary history. Understanding the computations of other-referenced information and representations of self and other will further inform how the brain was evolved to enrich what is often referred to as higher-level social cognition.

## Reinforcement learning framework

Reinforcement learning (RL) is perhaps the most influential framework developed to describe how an agent learns by interacting with its environment. RL is derived from the behaviorist view of animal behavior, in which an organism’s knowledge of the world is exclusively modeled based on its behavior. Crucially, RL theories focus on mechanistic accounts for behaviors based on several learning-related parameters established from empirical sources.

Both humans and nonhuman animals are excellent models for a variety of learning and decision-making tasks that are grounded on RL theories. Describing learning and learned outcomes through mathematical models is a powerful way to make explicit and testable predictions about how an organism will behave in a particular context and how they will make decisions that take into account internal states, such as motivation and subjective value.^27^ The RL framework can capture seemingly complex behaviors with relatively simple yet elegant rules, as in the famous Rescorla–Wagner model.^28^ Although various RL models differ in how they describe different cognitive phenomena, they share several core elements, such as the rate of learning or the salience of stimuli, to fit the specifics of learning and decision-making processes.

RL has its roots and applications in both engineering and psychology. RL has its core foundations in the work of Richard Bellman, most famous for developing the Bellman optimality equation and dynamic programming. The more widely appreciated root of RL is conceptualizing how organisms gather information from their environment to learn and make decisions. RL requires an agent that moves through different states, or contexts, in a given environment. Other necessary components include a reward signal, a value function, and a policy. Reward outcome is central to all forms of RL and consists of a quantity the agent gets as a result of its actions within the environment. The agent then computes a value function using that reward outcome that calculates the expected value of certain states/contexts as well as the conjunction of specific states and actions. The agent uses these value functions to develop a set of preferred actions, known as a policy. A model of the environment is an optional component of RL that can provide the organism with guidance on how to move from state to state.

In dynamic programming, developed by Bellman for engineering applications, a complete model of the environment is required. This idea requires the action of an agent to be guided by the expected payoff of the action in addition to the total expected payoff of potential actions in hypothetical future states.^29^ The same principle applies to temporal discounting (TD) models, the predominant form of RL model applied in psychological studies of humans and other animals.^30^ TD learning notably differs from dynamic programming, as it does not require any model of the environment. Instead, learning is accomplished by comparing expected reward to actual reward after a certain transition in time. This difference is the reward prediction error. This prediction error is used to update the value function and, ultimately, the policy of an agent interacting with its environment. Prediction error signaling is indeed the fundamental attribute of the original models of learning.^28^ In simple terms, a prediction error calculates the difference between what the animal expects to have happen and what actually happens to the animal on a given event or trial.^31^ This can also be described as an error signal.^32^

## Predictive coding and reinforcement learning in the brain

Prediction errors are effectively used as the signal that drives self-referenced learning. Organisms update their behavior on a trial by trial basis to account for new information provided by this discrepancy in expectation and outcome. In particular, the reward prediction error, which calculates the difference between expected payoff and received payoff, has been established as the striking correlate of a mathematical learning rule in neurobiology.^33^

A classic type of reward prediction error encoded in the brain is consistent with the type required for TD learning.^34^ Owing to the essential nature of reward to adaptive behavior, areas that encode reward are some of the best studied regions of the brain besides the regions of the brain involved in sensorimotor transformations. Classically, the dopaminergic substantia nigra^35^ and the ventral tegmentum^36^ as well as the dorsal and ventral striatum^37^ have been shown to be primary areas that process reward receipt and valuation, with dopamine’s relationship to reward now known as one of the most iconic behavior to neurotransmitter associations.^38^ As one might expect, these areas provide strong examples of encoding reward prediction errors.^39, 40^

Reward prediction error signals have also been found elsewhere in the brain. Primate lateral habenula neurons encode reciprocal information about reward outcomes to the previously described dopamine neurons in the midbrain.^41^ Notably, the activity of the lateral habenula neurons precede the activity of the dopamine neurons, suggesting that the lateral habenula neurons serve as an input for the prediction error signal detected in the midbrain.^41^ Furthermore, functional magnetic resonance imaging (fMRI) in humans has revealed the presence of multiple kinds of prediction errors and other learning-related signals across many reward-related structures in the cerebral cortex,^42–44^ indicating that the prediction error signaling is a widely generalized mechanism linking learning and decision-making. Applying these models to conceptualizing behavior and neural activity has proved fruitful in the study of learning and decision-making, perhaps most famously in the finding that midbrain dopamine neurons represent the TD reward prediction error.^33^

At least two important branches of research into RL in the neurosciences continue today. The first involves the potential balance between neural substrates of model-free (basic TD learning) vs. model-based (akin to dynamic programming) learning.^44^ These studies have collectively identified neural substrates of the model-based state transition error,^45^ representation of model-based in addition to model-free prediction error in the striatum and ventromedial prefrontal cortex,^46^ as well as brain areas that act as arbiters between model-free and model-based approaches.^47^ The second branch is vicarious reinforcement, which can also be modeled in a RL framework to account for how other’s behaviors could be used to update our own learning and decision-making processes using vicarious classes of prediction errors.^48^ RL can potentially be implemented in social learning about the actions and rewards of others.^48–50^

Such vicarious reinforcement in an RL framework would intuitively have to be performed in a model-based manner, as it is unclear how a model-free RL system could possibly learn about another agent without creating and updating a model of the other agent’s potential thoughts and future actions. Accordingly, research into how humans may use RL mechanisms to learn and make inferences about others have used a modified Q learning framework that involves a simulated other.^50^ Still, although RL constitutes a strong opportunity to explain and conceptualize social learning, there exist other computational frameworks that may be applied to social cognition. For example, some have argued that the putative TD reward prediction error forming the basis of RL theories may instead be interpreted in terms of expectation violation or even salience, especially in relation to activity in cortical regions.^51, 52^ Other models specifically designed to elucidate mentalizing via game theoretic approaches have been highly successful in exploring social behaviors in the relative absence of an explicit RL framework. These mainly consist of algorithms that produce iterative representations of other agents recursively ad infinitum.^53, 54^ Such approaches have not only explained typical human behavior in a stag-hunt game, but have also identified specific deficits in recursive social cognition in patients with autism spectrum disorders.^55^

Prediction error signals can occur for a variety of different events to be learned about, like action values, reward value, and reward timing.^40, 56, 57^ Furthermore, prediction errors are not limited to the reward domain. Evidence of prediction error calculations are even present in sensorimotor areas of the brain that deal with fine tuning actions like the cerebellum and the frontal eye fields^31^ (see Table 1 for types of prediction errors and associated brain regions). Therefore, a critic signal is responsible for correcting behavioral outputs and cognitive representations across a variety of functional domains of the brain, endorsing the notion that predictive coding is a key feature of the brain.

**Table 1:** Representative list of brain areas in which signals that can be described as prediction errors have been found from either primate electrophysiology or human neuroimaging studies

Prediction error computed	Correlated brain area	
Egocentric		
Self action	SC^31^	OFC^31^
(action executed)—(action	Cerebellum^31^	ACC^31^
intended)	FEF^31^	MCC^85, 86^
	LIP^31^	
	dlPFC^31^	
Self reward outcome	VTA^33, 34, 36, 57^	LHb^41^
(actual reward outcome)—(expected reward outcome)	VS/other striatum^31, 56, 84^	ACC^86^
	SN^35, 56^	dlPFC^31^
Self reward value	vmPFC^40^	
(actual value of reward)—(expected	VTA^40^	
value of reward)	SN^40^	
Self reward timing	VTA^35, 57^	
(actual timing of reward)—(expected timing of reward)	SN^35, 57^	
Allocentric		
Other action	dlPFC^84^	VS/other striatum^63–68^
(other’s actual action)—(other’s expected action)	dmPFC^91^	
Other reward outcome	ACCg^48^	MTG^42^
(other’s actual reward)—(other’s	vmPFC^84^	STS^42^
expected reward)	dmPFC^42^	TPJ^42^
Other motivation	ACCg^48^	
(other’s actual motivation)—(other’s expected motivation)		

This is not a comprehensive list but rather a list to highlight the presence of predictive coding in the brain. Note that the list for the action-related error signals is mostly adapted from Schultz and Dickinson^31^ review

*ACC* anterior cingulate cortex, *ACCg* anterior cingulate gyrus, *dlPFC* dorsolateral prefrontal cortex, *dmPFC* dorsomedial prefrontal cortex, *FEF* frontal eye field, *LHb* lateral habenula, *LIP* lateral intraparietal area, *MCC* middle cingulate cortex, *MTG* medial temporal gyrus, *OFC* orbitofrontal cortex, *SC* superior colliculus, *SN* substantia nigra, *STS* superior temporal sulcus, *TPJ* temporoparietal junction, *vmPFC* ventromedial prefrontal cortex, *VS* ventral striatum, *VTA* ventral tegmental area

As strides in describing increasingly complex human behaviors have been made, attempts to carry the study of learning and decision-making for the self into learning and decision-making that takes into account the behavior of others is now a subject of intense interest. Reacting appropriately to conspecifics and correctly anticipating their behavior is a necessity for social organisms, requiring them to rely on understanding each other just as much as they rely on understanding where to forage for food to survive. As expected, learning about others and representation of self and other are mediated by several reward-related brain structures.

## Neural basis of self-referenced and other-referenced reinforcement signals

In this section, we discuss selected research findings that have provided novel insights into how the brain signals self-referenced and other-referenced information in the domain of reinforcement learning and decision-making. When applicable, we focus on other-referenced prediction error signals regarding actions and reward outcomes relevant to reward-guided social learning.

### Striatum

Recent advances in the field of neuroscience have elegantly provided various supports for the use of RL mechanisms of learning about others. Although the striatum has long been a center of focus for self-referenced reward information and prediction error in the brain, the role of striatum in learning is not restricted to self-referential processing. In a study examining observational learning and vicarious reinforcement with respect to dopamine release, observer rats vocalized more and experienced significantly more dopamine release in the ventral striatum when seeing another rat receive reward compared to when reward was delivered to an empty box.^58^ These results extend the role of dopamine release in associations with prediction error signaling to the social domain, implicating the involvement of the similar RL mechanisms for signaling other’s reward outcome. Notably, the degree of dopamine release for other’s reward outcome was still substantially weaker compared to one’s own reward, suggesting that similar mechanisms are utilized but in ways that could be differentiated for self and other.^58^ In monkeys engaged in a task environment involving actions from and reward outcomes for self and other, neurons in the striatum signal one’s received reward but not the reward received by others while signaling the actions performed by others,^59^ indicating that there may be specializations for signaling self-referenced and other-referenced information in the striatum, and this differentiation may further depend on the encoding of action and reward outcome of another individual.

There is also evidence that the striatum represents other-referenced reward and prediction errors from human fMRI studies. When socially evaluated by peers, previous positive social interaction with an individual led to that individual being associated with positive outcomes, which correlated with activity in the striatum as well as the orbitofrontal cortex. This suggests that social interaction can similarly activate brain regions that typically signal reinforcing values of primary reinforcers.^60^ The striatum also appears to be involved in the relative valuation of reward where other’s performance is compared to one’s own performance.^61, 62^ In an ultimatum game where subjects give money to a partner and receive a proportion of it back, activation of the striatum was also correlated with prediction errors that reflect the difference between the offer the subject received from the partner and what they expected the partner to give, but not between how the subject expected to feel and how they actually felt, which appears to be reflected in ventromedial prefrontal cortex (vmPFC) and the posterior cingulate cortex.^63^

Furthermore, RL-like prediction errors regarding expectations formed about how others viewed the subjects were correlated with activity in the striatum, OFC, rACC, and anterior insula.^60^ A variety of economic-game style tasks that require learning about other’s actions and outcomes and/or modeling the internal states of others have reported that the striatum is implicated in these processes. For example, the observed actions of others influence one’s own economic decisions and this is reflected in striatal BOLD response.^64^ Furthermore, if added payoff for social learning is removed, so that only pure observation of others is necessary for the task, an interpersonal prediction error still occurs in the striatum.^65^ Similarly, there is evidence from a reciprocity game that learning to trust or not trust others based on their behavior is mediated by a prediction error signal in the caudate nucleus.^66^

Interestingly, these other-referenced prediction errors in the striatum may even be associated with social norms, given their activation in economic games that rely on feedback from others. A prediction error type signal associated with going against group opinion also has been shown to correlate with how subjects changed their behavior to conform with the group on subsequent judgements.^67^

In a trust game where an investor gives money to a trustee who can return a proportion of the money, the difference between the trustee’s repayment ratio expected by the actor and what the trustee actually repaid resulted in a prediction error in the striatum in the subjects who relied on the behavior of the partner for learning.^68^ In addition, in the same study, the difference between the investment ratio and the investor’s model of the other’s model of what the investor will do formed a second order prediction error. Notably, the study found that a subject who failed to deeply model the mind of the partner experienced more striatal correlates of the first type of prediction error (i.e., relying more on the action of the other), whereas the more a subject modeled the mind of the partner, the more likely they were to activate the striatum for the second order prediction error (i.e., relying more on the mental representation of the other).

### Anterior cingulate cortex

**Fig. 1:**
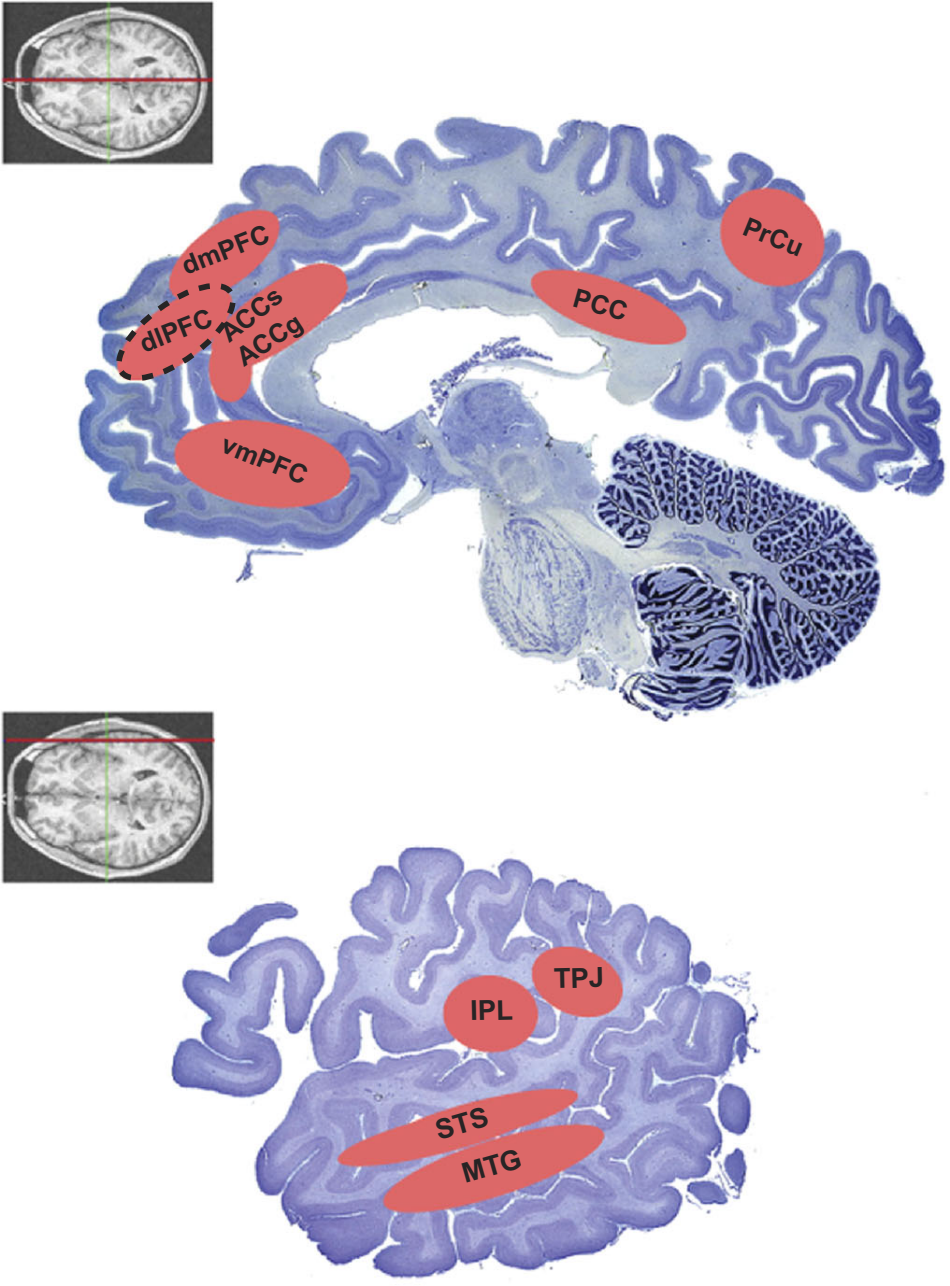
Key brain regions involved in representing information with respect to another individual. These areas are often implicated in mentalizing, detecting the beliefs of others, or signaling decision variables concerning another individual. See texts for how these areas are implicated in representing information with respect to another individual. The *insets* with coronal magnetic resonance images indicate the sections (*red line*) that correspond to the Nissl-stained sagittal slices. The *dotted outline* around an area indicates that this area is projected medially from the lateral surface for the purpose of including the area on a more medial aspect of the brain. Adapted with permission from http://www.brains.rad.msu.edu, http://brainmuseum.org, supported by the US National Science Foundation and the National Institutes of health. *ACCg* anterior cingulate gyrus, *ACCs* anterior cingulate sulcus, *dlPFC* dorsolateral prefrontal cortex, *dmPFC* dorsomedial prefrontal cortex, *IPL* inferior parietal lobule, *MTG* medial temporal gyrus, *PCC* posterior cingulate cortex, *PrCu* precuneus, *STS* superior temporal sulcus, *TPJ* temporoparietal junction, *vmPFC* ventromedial prefrontal cortex

The anterior cingulate cortex (ACC) is implicated in a variety of behaviors and cognitive states,^48, 69–71^ and could be summarized as an integrative area that relates to motivation and initiating reward-guided or goal-directed behaviors. Seen in this light, ACC may be a core locus of integrating different streams of self-referenced and other-referenced information for generating an adaptive action plan (see Fig. 1 for visualization of other-referenced reward and action areas of the brain). This is bolstered by the considerable evidence that ACC is engaged during social decision-making, with its neuronal signals reflecting information processing about self, other, or both.^48, 72–77^ In the domain of observational learning, the ability of mice to learn by observing the shock of conspecifics can be effectively abolished by ACC-specific deletion of calcium currents.^78^ Relatedly, observational aspects of pain have been a major focus for investigating empathy in the human brain. Observing a demonstration of another person being injured and experiencing pain elicits empathic concerns and actively engages a specific portion of the ACC that is also similarly activated when experiencing pain.^79^ Such shared mechanisms support that observation-driven vicarious pain processing was co-opted or repurposed from processing one’s own pain.

ACC may represent a critical junction in the cortical pathway of representing and differentiating self and other through processing motivation from the perspectives of self and others.^48^ Monitoring the spiking activity of individual ACC neurons while monkeys played a social reward allocation task, in which an actor animal has an option to deliver or withhold juice rewards to and from the recipient,^80^ showed that there are specializations with respect to signaling the reward outcome of self and other. More specifically, in the gyrus of ACC (ACCg), some neurons exclusively encode self reward, whereas others exclusively encode other’s reward, and still some encoded the reward outcomes of self and other.^81^ Notably, lesioning ACCg, but not the ACC sulcus (ACCs), abolishes social valuation in monkeys,^82^ indicating a causal contribution of ACCg in social cognition. Similarly, in the human brain, the rostral ACC neurons, overlapping with the ACCg neurons mentioned above, signal-reward outcomes from others during a card game requiring observational learning.^77^

Furthermore, neurons in ACC have been shown to mediate collective reward-guided actions when monkeys play a prisoner’s dilemma game,^74^ providing strong evidence that self and other processes are integrated in ACC. The evidence of self and other integration in ACC is also supported by the presence of an anatomical gradient along the human cingulum mapping self and other in a trust game that is absent without a responding partner.^83^ Moreover, it has been postulated that the ACCs and the ACCg represent distinct streams of information.^18, 48, 72, 82^

**Fig. 2:**
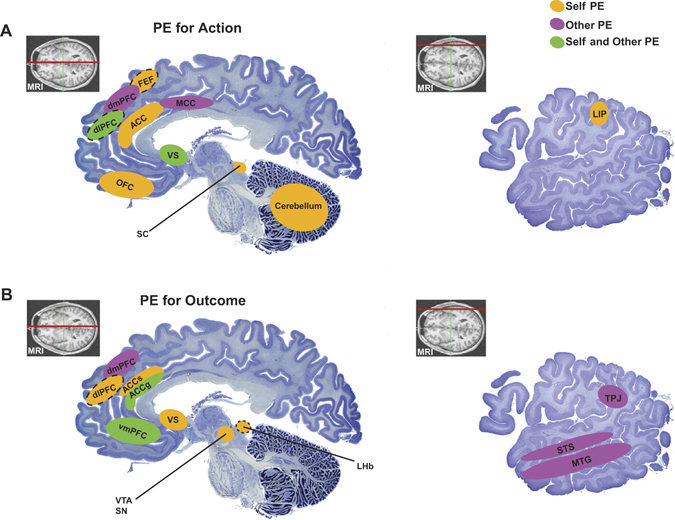
Key brain regions that have been shown correlates of self-referenced prediction errors (in *yellow*) or other-referenced prediction errors (in *purple*), or both kinds of prediction errors (in *green*) in the domain of actions (**a**) and reward/value outcomes (**b**). For motor learning-related errors, we only provide representative areas as they are beyond the scope of this review. It is worthwhile to note that the distributions of these areas for their involvements in self-referenced or other-referenced prediction error signaling are naturally constrained by the amount of research examining different types of prediction errors. The *insets* with coronal magnetic resonance images indicate the sections (*red line*) that correspond to the Nissl-stained sagittal slices. The *dotted outline* around an area indicates that this area is projected medially from the lateral surface for the purpose of including the area on a more medial aspect of the brain. Adapted with permission from http://www.brains.rad.msu.edu, http://brainmuseum.org, supported by the US National Science Foundation and the National Institutes of health. *ACC* anterior cingulate cortex, *ACCg* anterior cingulate gyrus, *ACCs* anterior cingulate sulcus, *dlPFC* dorsolateral prefrontal cortex, *dmPFC* dorsomedial prefrontal cortex, *LHb* lateral habenula, *LIP* lateral intraparietal area, *MTG* medial temporal gyrus, *OFC* orbitofrontal cortex, *SC* superior colliculus, *SN* substantia nigra, *STS* superior temporal sulcus, *TPJ* temporoparietal junction, *vmPFC* ventromedial prefrontal cortex, *VS* ventral striatum, *VTA* ventral tegmental area

Accurate social learning requires multiple types of prediction error signals with respect to others (see Fig. 2 for representation of self-referencing and other-referencing prediction errors in the brain). For example, observational action prediction errors signal the difference between the actual action of the other and the expected action, whereas vicarious outcome prediction errors signal the difference between the actual and the predicted outcome of the other.^48, 84^ Furthermore, to estimate the motivation of others, vicarious dynamic prediction errors signal the difference between the actual and estimated movement kinematics of others during their actions.^48^ Prediction errors for self-referenced action values have been reported in ACC,^85, 86^ and both the sulcus and gyrus portions of the ACC are implicated in self-reward valuation and decision-making.^87, 88^ The ACCs is most well-studied for involved in multitudinous functions, from error detection and motivation to cognitive control and response selection.^85^ Recently, there is an extensive debate over whether or not ACCs are involved in computing value-guided behavioral adaption or cognitive control.^69–71^

Notably, there seem to be functional dissociations for signaling self-referenced and other-referenced information between the gyrus and sulcus. For example, prediction errors related to the choices made by another person are found in the ACCg but not in the ACCs.^48, 89^ Furthermore, the ACCs neurons encode reward outcomes in self-referenced manner in a social decision-making task, whereas a sub-group of the ACCg neurons do so in an other-referenced manner.^72^ Similarly, in a competitive game, self-referenced reward outcome prediction errors correlate with activity in the ventral striatum, but, critically, belief-based prediction errors about the competitive partners action are encoded in rostral ACC (rACC).^90^ Furthermore, in a social decision-making task involving utilizing advice from another person, learning rates for self and other are differentially computed by the ACCs and the ACCg, respectively.^49^ Overall, although social signals have been detected in ACC, the ACCg is most clearly linked to other-referenced information processing based on accumulating evidence spanning whole-brain neuroimaging, electrophysiological recording, and anatomical specializations.^48^

### Prefrontal cortex

The prefrontal cortex has many subsections, and is often thought of as the locus of higher level cognitive processes related to decision making. It is intuitive, then, many parts of the prefrontal cortex process other-referenced information. When observing erroneous choices by another individual informs an association between a specific target and a possible reward during a turn-taking decision-making task in pairs of monkeys, neurons in the dorsomedial frontal cortex encode the errors made by the partner monkey, serving a social error monitoring function,^91^ which relies on other-referenced information. Similarly, the vmPFC encodes in humans the value of observing another person’s behavior in a reward seeking task, and correlates with that individual’s move towards conforming to social norms.^92^ Other types of prediction errors are also found in the prefrontal cortex. When participants learn the contingencies between stimulus and reward outcome through direct experience or observing the action and outcome of another person, different reward-related prefrontal structures signal learning-related events for self and other. In such scenarios, the ventral striatum signals self prediction errors, the dorsolateral prefrontal cortex (dlPFC) signals other’s action prediction errors, and vmPFC signal other’s outcome prediction errors.^84^

Furthermore, Suzuki et al.^50^ examined the neural correlates of learning stimulus-reward outcome contingencies when participants learned the association directly and when the participants predicted which stimulus another person will likely choose, encouraging the participants to model or mentally simulate the other individual. This manipulation necessitated the use of an other-referenced prediction error, one that calculates the discrepancy between what the other person does and what the participant thought the other person would do. Again, different parts of the prefrontal cortex are engaged as a function of self-referenced and other-referenced computation. The vmPFC tracked the simulated other’s prediction error in a similar manner to self, whereas the simulated other’s action prediction error was signaled by the dorsomedial prefrontal cortex (dmPFC) and dlPFC.^50^ Notably, neuronal activity in the monkey dmPFC has been shown to closely reflect the strategy of an opponent in a competitive reward-based task, further strengthening the specialized role of dmPFC in simulating others.^93^ This is consistent with the findings of Behrens et al.,^49^ in which separable reward signals were computed in ACCg and ACCs for other- referenced and self-referenced reward information, respectively, and that these signals were integrated in vmPFC.

Finally, the orbitofrontal cortex (OFC) is a key cortical region for signaling reward value^94^ and is also associated with showing value prediction error signaling.^95^ Although OFC neurons are sensitive to social reward context involving self and other,^96^ the reward outcome encoding of these neurons seems to be self-referenced,^72^ suggesting that OFC may be more restricted to mediating behavioral adaptations, including adjusting to social context, in a self-referenced framework.

Encoding of various prediction errors regarding others is a signature of many reward-related regions of the brain, suggesting a tight biological link between self learning and learning about or from others. In particular, these findings endorse the notion that comprehending and learning from the experience of another person is processed under shared predictive coding principles with particular regional specializations for the self and other domain.

### Temporal parietal junction and mentalizing networks

When ToM is engaged, demanding the modeling of another individual, precuneus (PrCu), posterior cingulate cortex (PCC), as well as superior temporal sulcus (STS), temporal parietal junction (TPJ), and the medial prefrontal cortex (mPFC) are particularly activated over others.^97^ STS and TPJ have long been considered as the neural hotspots for higher-level cognitions like ToM and modeling the minds of others. TPJ, in particular, has been regarded as a uniquely social cognition-focused area,^98^ with evidence that TPJ is necessary for representing the belief of others.^99^ A meta-analysis of ToM-related areas determined that the most reliably implicated areas are TPJ and mPFC, with activations in PrCu and STS being sensitive to the types of ToM engaged in the context of various ToM measures.^100^ Notably, researchers have found a close link between self-referential thoughts and mentalizing of others in mPFC,^101^ indicating how self-referenced and other-referenced information is associated with one another in one of the key regions of the mentalizing network.

Notably, recruitments of TPJ and STS are not specific to tasks designed to measure ToM. TPJ and STS are also activated in situations when considering other’s information to guide one’s actions, suggesting their involvements in broadly defined other-referenced computation. When participants take into account the advice of another person to make a decision about obtaining potential rewards, dmPFC, middle temporal gyrus (MTG), STS, and TPJ activations signal social prediction error.^49^ Furthermore, when playing a simplified poker game against a human opponent and a computer algorithm, TPJ emerges as a unique region for predicting social decisions that are behaviorally relevant.^102^ In addition, STS has been well known for their roles in social perception from visual cues.^103^ Therefore, tracking and interpreting socially relevant information may be the fundamental building blocks of these areas constituting a so-called mentalizing network. Recently, an elegant proposal was put forward suggesting that TPJ is a computational hub in which distinct cognitive processes, like attention, memory, sensory perception, and language all converge together to generate a representation of behaviorally relevant social context.^104^

Corresponding to this idea, many of the nodes in this proposed mentalizing network have been observed to perform additional functions potentially relevant to other aspects of social behavior. For example, PCC has been proposed to compute subjective value^105^ as well as other-related social processes including person perception, person updating, and first impressions.^106–108^

## Concluding remarks

Hale and Saxe^109^ have proposed that mentalizing may be a fundamentally predictive process. Although our current understanding of how the brain implements processes described in theory–theory or simulation theory is not complete, the fact that other-referenced prediction errors appear to be represented neurally suggests that there are shared prediction-based learning mechanisms for social learning and reinforcement learning. The neural mechanisms underlying other-referenced learning may be co-opted from the predictive mechanisms used to learn for the self, one of which is prediction error signaling. Connecting the terminology of reinforcement learning and decision making to the social domain can enhance the development of ideas and methods in studying how we think about others.^110^

There are many additional dimensions of other-referenced learning that remain to be explored. As experimenters continue to push the limits of studying social learning, interaction, and valuation; we may find ourselves brushing up against the limits of how the brain operationalizes what is “social” and “nonsocial”. Beyond other-referenced representations in the brain, social processing can also refer to the comparison of social agents and nonsocial, yet interactive, agents. Although divergent brain areas may apply similar computations to account for self and other, the neural processes underlying social information processing may not be categorically distinct from other types of information, but rather lie on a continuum. For example, when human participants play a game with other individuals or slot machine partners programmed to display varying levels of generosity, activations in TPJ, PCC, PrCu, vmPFC, and several other regions reflected the prediction errors for generosity similarly for both human and slot machine partners.^111^

This finding and many others that have observed modulatory differences in brain activations between social and nonsocial information may suggest that the brain may not in fact differentiate the two categorically but processes information as a function of implementing algorithms demanded by specific behavioral constraints. Perhaps social functions could be regarded as repurposed ancestral functions of the brain evolved to deal with an organism’s social environment.^112^ Then the notion of the “social brain” should be concerned with how specific sets of commonly used computational algorithms are utilized to guide adaptive behaviors.
